# Supplementary material for: Denoising-autoencoder-facilitated MEMS computational spectrometer with enhanced resolution on a silicon photonic chip
Source: Nat Commun. 2024 Nov 26;15:10260. doi: 10.1038/s41467-024-54704-1 (PMC11599558; doi:10.1038/s41467-024-54704-1)
Supplement: Supplementary file 1 — Supplementary Information [file 41467_2024_54704_MOESM1_ESM.pdf]

Supplementary Information

Denoising-autoencoder-facilitated MEMS computational spectrometer with enhanced resolution on a silicon photonic chip

Jing Zhou[1,2,#], Hui Zhang[3,4,5,6,#], Qifeng Qiao[7], Heng Chen[1,2], Qian Huang[1,2], Hanxing Wang[1,2], Qinghua Ren[1,2], Nan Wang[1,2], Yiming Ma[1,2,*], and Chengkuo Lee[8,9,10,*]

[1]School of Microelectronics, Shanghai University, Shanghai 201800, China

[2]Shanghai Collaborative Innovation Center of Intelligent Sensing Chip Technology, Shanghai University, Shanghai 201800, China

[3]Institute of Precision Optical Engineering, School of Physics Science and Engineering, Tongji University, Shanghai 200092, China

[4]MOE Key Laboratory of Advanced Micro-Structured Materials, Shanghai 200092, China

[5]Shanghai Institute of Intelligent Science and Technology, Tongji University, Shanghai 200092, China

[6]Shanghai Frontiers Science Center of Digital Optics, Shanghai 200092, China

[7]Shanghai Industrial μTechnology Research Institute (SITRI), Shanghai 201800, China

[8]Department of Electrical and Computer Engineering, National University of Singapore, Singapore 117583, Singapore

[9]Center for Intelligent Sensors and MEMS (CISM), National University of Singapore, Singapore 117608, Singapore

[10]National Centre for Advanced Integrated Photonics (NCAIP), Singapore 639798, Singapore

[#]These authors contributed equally: Jing Zhou and Hui Zhang

[*]Correspondence and requests for materials should be addressed to (Y. Ma) yimingma@shu.edu.cn and (C. Lee) elelc@nus.edu.sg

Supplementary Note 1

Polynomial fitting for **Δ*n***: To investigate the effective index difference Δ*n* as a function of the wavelength (λ) and the vertical coupling gap (h) between the two waveguides, we simulate the waveguide modes using Lumerical MODE solver. The width and thickness are 350 and 220 nm, respectively, for both waveguides. The lateral coupling gap between the two waveguides is kept at 200 nm. The effective index difference Δ*n* is calculated with the vertical gap varying from 0 to 0.6 μm in the 1.3-1.6 μm wavelength range. As shown in **Fig. S1**, the simulation results can be fitted well using the polynomial approximation given by **Eq. 3** in the main text with the R-squared value as high as 99.72%.

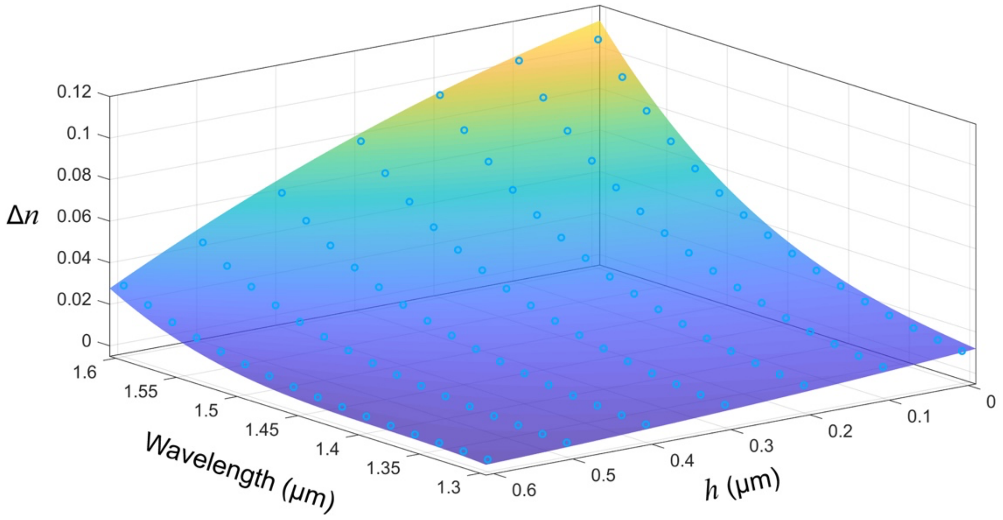

Fig. S1 Model fitting of the effective index difference Δ*n*. The simulation results are shown in blue circles and the fitted model is shown in surface.

Supplementary Note 2

Polynomial fitting for h: The relationship between the vertical coupling gap (h) of the waveguide coupler and the applied bias voltage (V) is investigated using COMSOL Multiphysics. The vertical coupling gap is simulated with the same bias voltage sequence setting as in the main text. The simulation results are well-fitted by the polynomial approximation given by **Eq. 4** in the main text, as demonstrated in **Fig. S2**. The R-squared value is as high as 99.95%. The electrostatic pull-in occurs at a cantilever tip displacement of ~45% of the initial actuator gap[S1]. The initial actuator gap equals the BOX layer thickness of 2 μm. Thus, the cantilever tip displacement, i.e., the vertical coupling gap h, is ~0.9 μm when the electrostatic pull-in occurs. The pull-in voltage is estimated to be ~34.3 V from the fitting curve in **Fig. S2**.

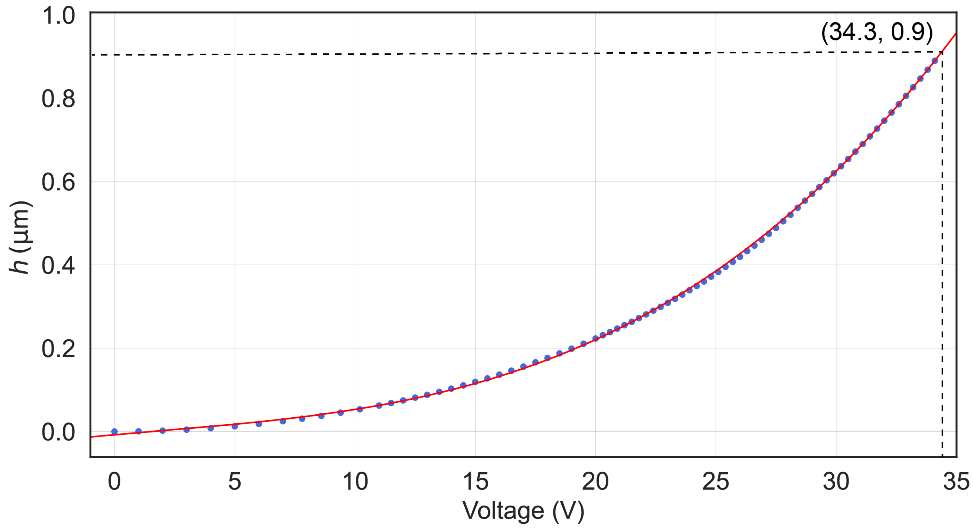

Fig. S2 Model fitting of the vertical coupling gap h. The simulation results and the fitted model are represented by the blue dots and the red curve, respectively.

Supplementary Note 3

Design innovations in MEMS spectrometer: Compared to our previous work[S2], there are five points of innovations in terms of working principle and device structure:

1) In the previous work, it was thought that the directional coupler needs to be modulated from the initial coupled condition to a fully decoupled condition to facilitate the spectral reconstruction. Such a coupling condition transition requires a large tuning displacement of the MEMS cantilever, which exceeds the tuning range that can be offered by the release of the BOX layer, with the electrostatic pull-in effect taken into account[S1]. As a result, a non-standard and low-yield flip-chip bonding process was needed to enable the large tuning displacement. Here in this work, we have first investigated the relationship between the device tuning range and the spectral reconstruction performance using correlation analysis (see more details on correlation analysis in **Supplementary Note 5**). The self- and cross-correlations of three tuning ranges (from the initial coupled condition to a weakly/moderately/fully decoupled condition) are studied. As shown in **Figs. S3a, b**, the moderately decoupled condition results in the smallest self-correlation width as well as considerably low cross-correlation. The fully decoupled condition can further decrease the cross-correlation, but at the expense of larger self-correlation width, thus tends not to improve the spectral resolution. The dual-wavelength spectra reconstruction results as shown in **Figs. S3c-e** confirm that the moderately decoupled condition offers the finest reconstruction resolution. The adoption of a moderately decoupled condition in our work not only guarantees a satisfactory spectral resolution but also reduces the required tuning range to a value that can be offered by the release of the BOX layer, i.e., below pull-in (see **Supplementary Note 2**), which largely simplifies the device configuration and fabrication process.

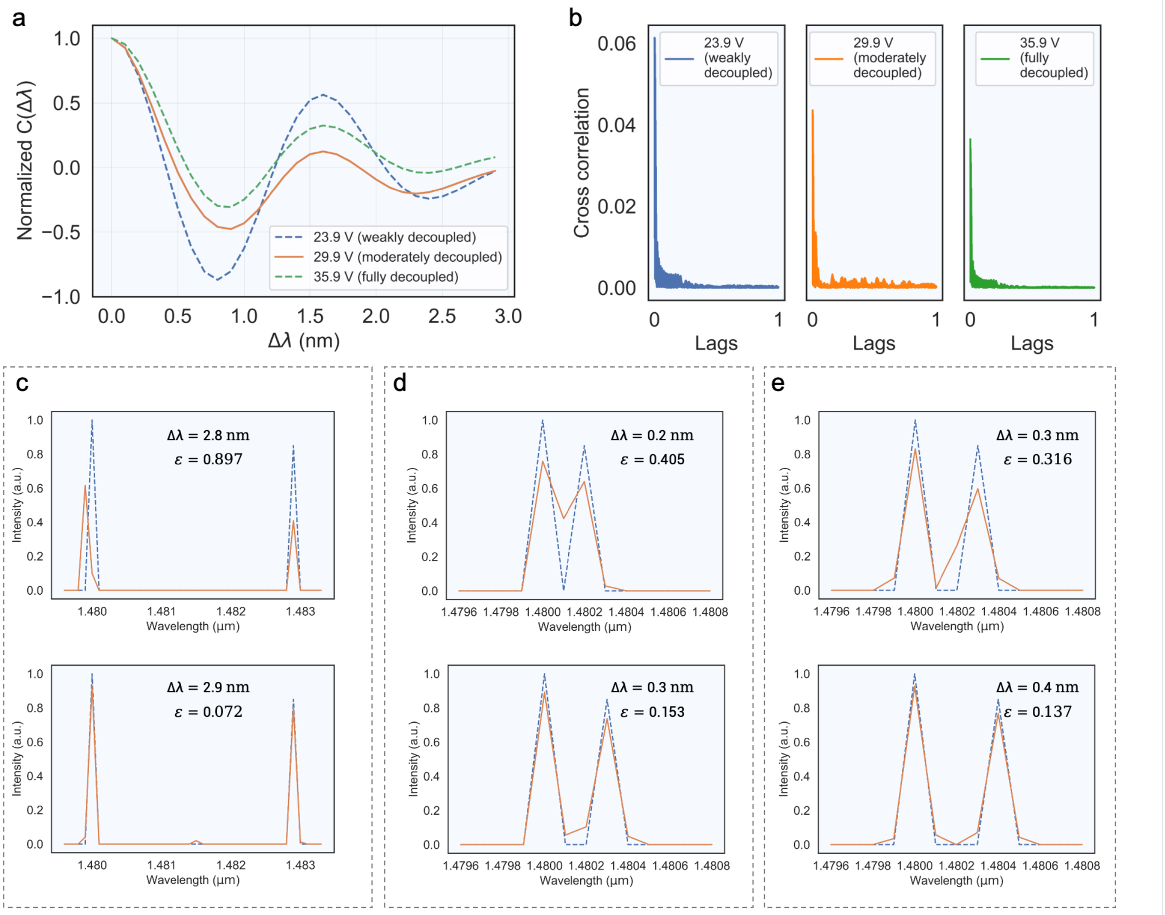

Fig. S3 Relationship between tuning range and reconstruction performance. **a** Self-correlation function and **b** Cross-correlation of three tuning ranges from the initial coupled condition to a weakly/moderately/fully decoupled condition. **c-e** Spectrum reconstruction results at the weakly/moderately/fully decoupled condition, respectively.

2) In the previous work, subwavelength grating (SWG) was employed as the supporting structure for the suspended waveguides in the mid-infrared. However, when migrating the SWG design to the telecommunication wavelengths, the minimum feature size is typically below 150 nm, which is challenging for common silicon photonics foundries[S3,4]. In this work, we employ and optimize a trapezoidal supporting structure for low-loss suspension of the waveguides, as shown in **Fig. S4**. Compared to SWG, the trapezoidal supporting structure possesses lower fabrication restriction and better wavelength scalability. We fix the beam width to be 0.4 μm, and then optimize the structural parameters of the trapezoid. With a height, baseline length, and topline length of 0.4, 8, and 3 μm, respectively, the insertion loss of the trapezoidal supporting structure is optimized to 0.098 dB, which is comparable with the reported elliptical supporting structure[S5]. The minimum feature size of 0.4 μm can be conveniently fabricated by common silicon photonics foundries.

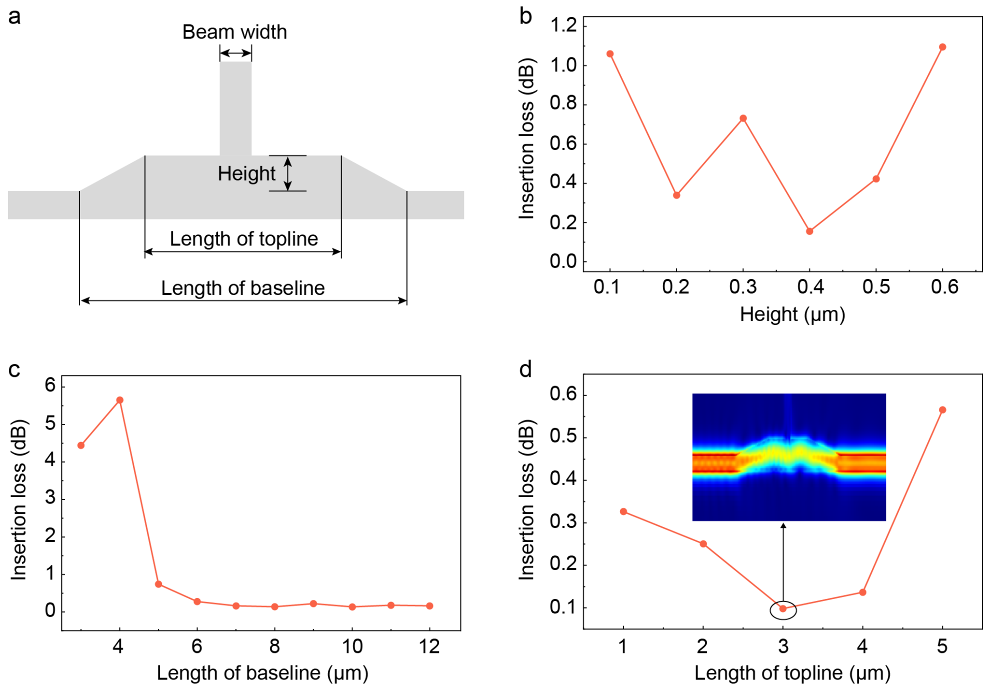

Fig. S4 Design and optimization of trapezoidal supporting structure. **a** Schematic illustration of structural parameters. **b** Optimization of the trapezoid height. **c** Optimization of the trapezoid baseline length. **d** Optimization of the trapezoid topline length, inset shows the electric field profile with the optimized structural parameters.

3) Unlike the normal directional coupler employed in the previous work, here we simplify the bus waveguide to a straight waveguide, which reduces the propagation loss and improves the SNR.
4) Unlike the grating couplers employed in the previous work, adiabatically tapered edge couplers are used to facilitate the fiber-to-chip coupling in our work, which significantly enlarge the device bandwidth. **Fig. S5** shows the transmission spectrum of the edge coupler, showing a low coupling loss of below 4 dB/facet across the wavelength range of 1.3-1.6 μm and a 1-dB bandwidth of ~250 nm.

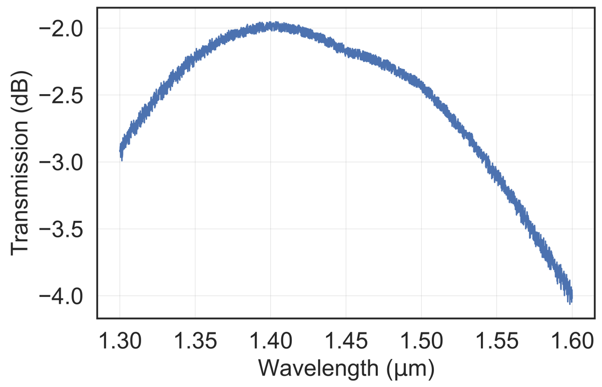

Fig. S5 Transmission spectrum of the edge coupler.

5) The residual stress in the Si device layer will induce significant unevenness of the cantilever and deteriorate the coupling between the two waveguides, limiting the achievable coupling length. Thus, the long coupling length was offered by two cascaded waveguide couplers in our previous work. Here in this work, we deposit an Al layer with proper length and thickness onto the Si cantilever to improve the stiffness and thus the flatness of the cantilever[S6]. Consequently, we are able to implement a long coupling length using a single waveguide coupler. The good flatness of the cantilever ensures uniform coupling along the whole coupling length, which is confirmed by the measured calibration matrix.

Supplementary Note 4

MEMS spectrometer properties: **Fig. S6** shows the static transmission spectrum of the whole device at the initial state. Our device shows a total insertion loss of 6.4-10.6 dB across the whole wavelength range of 1.3-1.6 μm. For the collection of the calibration matrix, wavelength-dependent features of laser intensity, edge coupler efficiency, and detector responsivity are cancelled out by taking the transmission spectrum of a straight waveguide on the same chip as a reference.

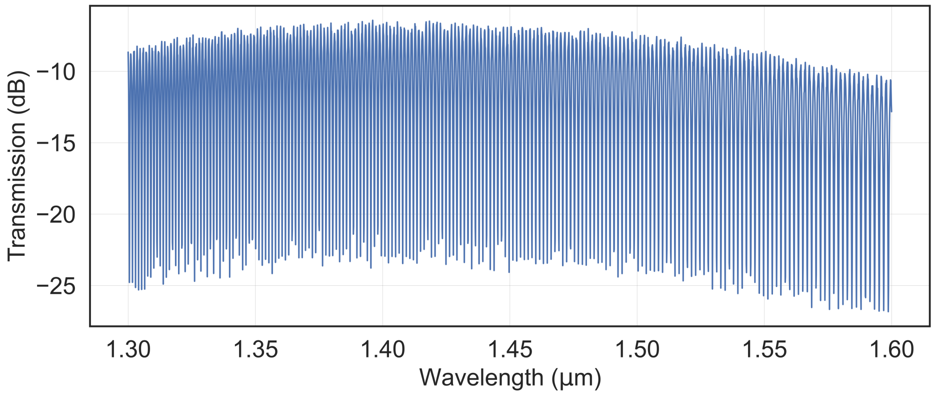

Fig. S6 Static transmission spectrum of the whole device at the initial state.

The modulation speed of the MEMS actuator is characterized by measuring its frequency response using a laser Doppler vibrometer, as shown in **Fig. S7**. The measured mechanical resonance frequency of 177 kHz agrees with the simulated response time of ~12 μs (see **Fig. S10d**).

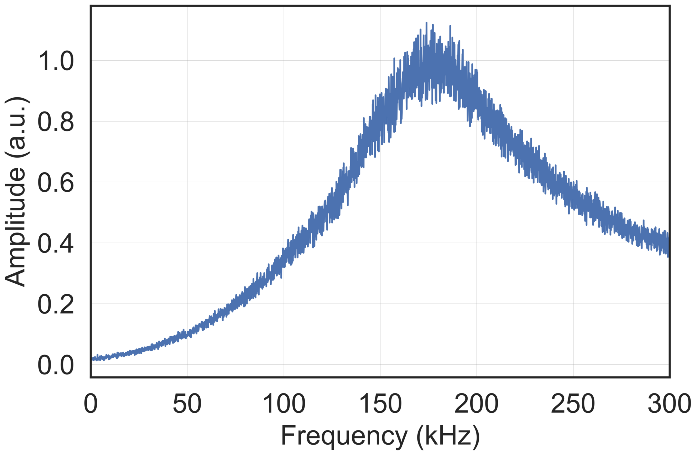

Fig. S7 Frequency response of the MEMS actuator.

Supplementary Note 5

Correlation analysis: The performance of a spectrometer is expected to achieve the two properties: (i) The spectral response at each sampling channel should have diverse features, so that the correlation length in the wavelength span should be small to provide high spectral resolution; (ii) The transmission spectra for any two sampling channels should be very different, i.e., orthogonal, to provide a transmission sampling matrix with large rank[S7]. Hereby, self- and cross-correlations are studied on the transmission sampling matrix to evaluate these two properties.

For the transmission sampling matrix, its row vector is referred to as spectral responses (at different channels), while its column vector is the temporal speckle at a single wavelength. Each channel's spectral response should contain adequate sharp features to provide high sampling resolution, thereby achieving high spectral resolution. The spectral resolution depends on the change in wavelength required to generate an uncorrelated speckle pattern, which can be quantified by the spectral self-correlation function:

$$C(\Delta\lambda, x) = \frac{\langle I(\lambda,x)(\lambda+\Delta\lambda,x)\rangle}{\langle I(\lambda,x)\rangle\langle\lambda+\Delta\lambda,x\rangle} - 1 \quad \text{(S1)}$$

where $I(\lambda, x)$ is the intensity recorded by the sampling channel x for input wavelength λ, and $\langle\cdots\rangle$ represents the average over λ. In our MEMS spectrometer, a small shift in the input wavelength will cause a significant change in the transmitted speckle pattern. The average of the computed $C(\Delta\lambda)$ of our spectrometer across different channels x, i.e., the $\langle C(\Delta\lambda, x)\rangle_x$, is depicted in **Fig. S8a**. C is normalized to 1 at $\Delta\lambda = 0$, and its half-width at half-maximum, namely self-correlation width $\delta\lambda$, is 0.28 nm, meaning that a wavelength shift of $\delta\lambda$ is sufficient to reduce the degree of correlation of the speckle pattern to 0.5. $\delta\lambda$ provides an estimation of the spectral resolution since it is impossible to resolve two wavelengths with highly correlated speckle patterns. The actual resolution also depends on the reconstruction algorithm and the experimental noise of the measurements. The decrease in resolution caused by noise is exactly the part that we would like to get rid of so that the resolution can be rectified to the optimal 0.28 nm, even in a noisy environment. Our proposed autoencoder scheme rectifies the resolution from 1.2 to 0.4 nm.

Since the nature of the computational spectrometer is to use much fewer equations to solve many unknown values, we would expect that each equation (sampling channel) is independent of the others. Ideally, the rank of the transmission sampling matrix needs to be as large as possible, in other words, the transmission spectra of different sampling channels have to be ideally orthogonal with zero cross-correlation. Here, we calculate the cross-correlation function

and the absolute value of the averaged cross-correlation between one specific sampling channel and all the other sampling channels (**Fig. S8b**). It can be seen that the spectra of these sampling channels contain diverse features with very little cross-correlation value, approaching almost 0, which proves the effectiveness of our designed time-domain modulation channels. Another direct visualization of the sampling channel dependencies is the Pearson correlation coefficient between the spectral responses of any two distinct sampling channels, as depicted in **Fig. S8c**. It is observed that the sampling channels all exhibit high linear independence, resulting in almost zero Pearson correlation coefficient between two spectral responses (**Fig. S8d**). In summary, our MEMS spectrometer satisfies the two criteria of low self-correlation and minimal cross-correlation, by numerical calculations using the transformation sampling matrix obtained from the MEMS spectrometer.

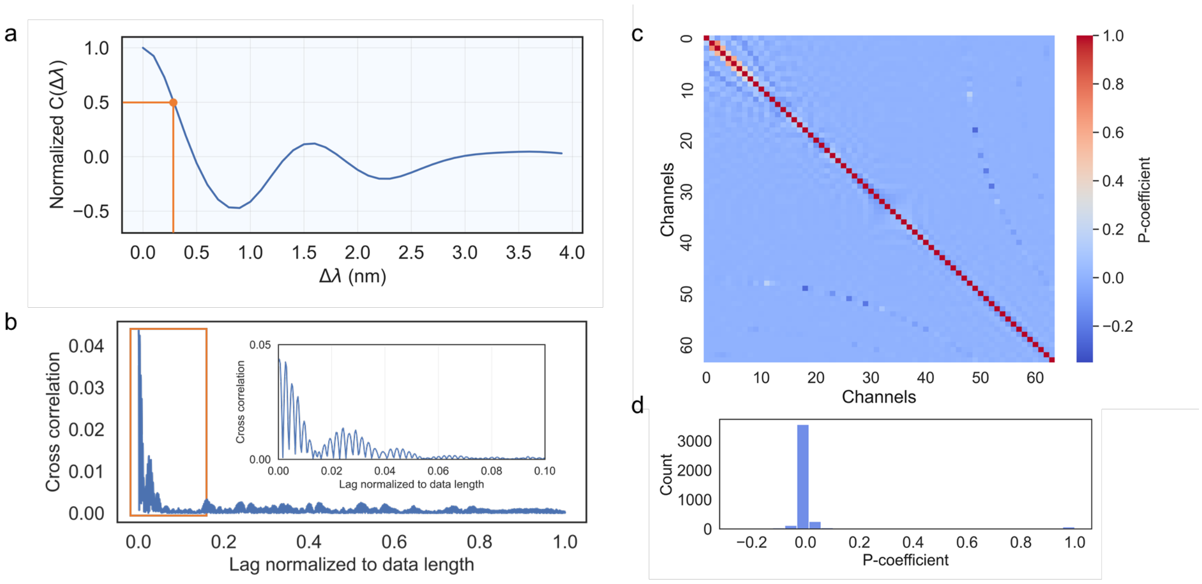

Fig. S8 Correlation analysis. **a** Spectral self-correlation function $C(\Delta\lambda)$ derived from the measured $I(x,\lambda)$ of our MEMS spectrometer. The half-width at half-maximum is 0.28 nm, indicating that a wavelength shift of 0.28 nm reduces the degree of spectral correlation by half. **b** Absolute value of the averaged cross-correlation between one specific sampling channel and all the other sampling channels. **c** Pearson correlation coefficients (P-coefficients) between the transmission spectra of each pair of sampling channels. **d** Histogram of the P-coefficient, with over 96% falling within the range of [-0.1, 0.1], suggesting a nearly zero linear independence.

Interferogram function: When we combine **Eqs. 3** and **5** from the main text, the output power is:

$$P_o(\lambda, V) = A(\lambda)\cos^2\left((a_1\frac{1}{\lambda} + a_2 + a_3\lambda)\pi L f_2\big(h(V)\big)\right) \tag{S2}$$

For each specific V, the spectra responses can roughly be simulated by $\cos^2\left(C(a_1\frac{1}{\lambda}+a_2+a_3\lambda)\right)$, where C is a constant value calculated by $\pi L f_2\big(h(V)\big)$.

Compared to traditional Michelson spectrometers (typically modelled as $\cos^2\left(C\frac{1}{\lambda}\right)$), our interferogram function (**Eq. S2**) includes the higher-order terms - this is because we have specially designed the waveguide dimensions to optimize the spectrometer's performance (resolution and matrix rank), as shown in **Fig. S9**. As observed, when the waveguide becomes narrower, both the self-correlation width and the cross-correlation decrease thanks to the more significant higher-order terms, resulting in better spectral reconstruction resolution and accuracy. For waveguide widths of 300 nm and lower, the optical mode cannot be well confined in the waveguide core and would lead to high propagation loss, which lowers the SNR and may worsen the reconstruction performance. Therefore, a waveguide width of 350 nm is chosen.

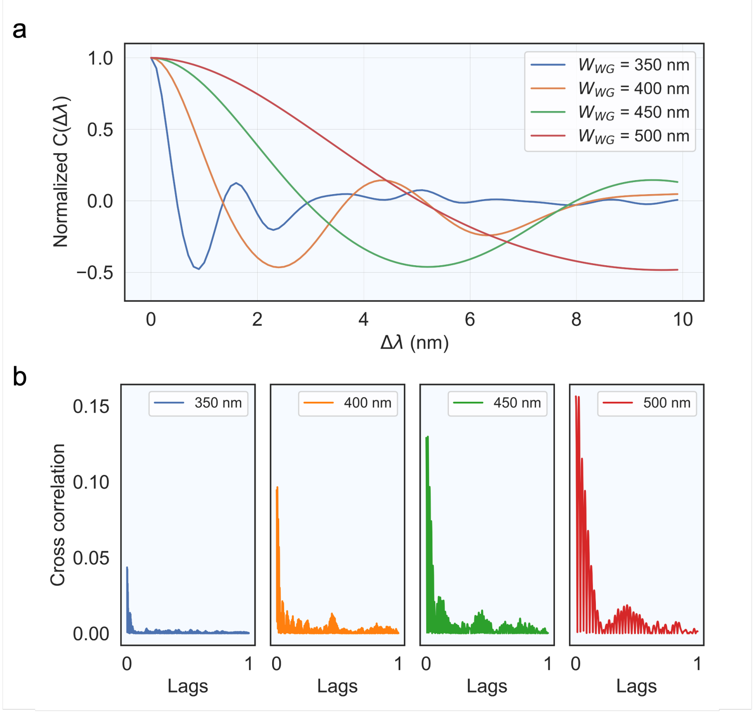

Fig. S9 The spectrometer's performance, i.e., **a** self-correlation function and **b** cross-correlation obtained from the transmission sampling matrix, with varying waveguide width. The $\delta\lambda$ for the four waveguide widths is 0.28, 0.81, 1.76, and 3.11 nm, respectively. The mean cross-correlation value for the four waveguide widths is 0.0010, 0.0040, 0.0080, and 0.0136, respectively.

Supplementary Note 6

Spectrum reconstruction method: The segmented regularization terms $\alpha\|\mathbf{R}_1+\mathbf{R}_2\|_1$ and $\beta\|\mathbf{R_2}\|_2^2$ in **Eq. 8** only set a *general range* of possible characteristics that may occur in a spectrum. It does not require specific knowledge of spectral contents before measurement, as most naturally occurring features can be covered by these terms. In addition, the hyperparameters α and β can be automatically optimized via cross-validation. The optimization process does not require any manual parameter selection. Using **Eq. 8**, it is feasible to attain the same level of reconstruction accuracy for diverse input spectra without any priori. Similar regularized iterative methods have been used in prior studies[S8–11].

To be specific, $\alpha\|\mathbf{R}_1+\mathbf{R}_2\|_1$ with L_1-norm provides sparsity regularization that compresses spectra into discrete lines, whereas $\beta\|\mathbf{R_2}\|_2^2$ with L_2-norm provides Tikhonov regularization that smoothens spectra into continuous bands. Generally, a spectrum is either discrete or continuous; therefore, the proposed segmented regularization terms can cover most naturally occurring spectral features. A mixed spectrum can also be rebuilt using this method with non-zero values for both α and β. By using cross-validation, these hyperparameters can be automatically optimized without any manual selection.

To further verify the effectiveness of the proposed modified regularization method, the solvability of **Eq. 8** is assessed with the Picard plot. Based on truncated singular value decomposition (SVD), the naïve solution to the inverse problem can be written as:

$$\mathbf{R}=\sum_{i=1}^{N_s}\frac{\mathbf{u}_{(i)}^{\mathrm{T}}\mathbf{I}}{\sigma_i}\mathbf{v}_i \tag{S3}$$

where $\mathbf{u}_i$ denotes the i-th left singular vector, $\mathbf{v}_i$ denotes the i-th right singular vector, σ_i denotes the singular value, and N_s denotes the sampling channel number. **Equation S3** can be interpreted as that the naïve solution of the input spectrum ($\widehat{\mathbf{R}}$) is formed on the basis of right singular vectors ($\mathbf{v}_{(i)}$) that are weighted by SVD coefficients ($\mathbf{u}_{(i)}^{\mathrm{T}}\mathbf{I}/\sigma_i$). Thus, to prevent an infinite integral and retrieve all spectral information, it is necessary to ensure that the SVD coefficient levels off to a finite value, also known as the Picard condition[S12]; otherwise, the integral of channels will be infinite and the iterative process will suffer from a poor convergence. Four distinct types of spectra (**R**) are used for testing: plateau, Gaussian, spike, and random functions, as shown in the first row of **Fig. S10a**. **Figures S10b, c** show the calculated absolute values of SVD coefficients ($|\mathbf{u}_{(i)}^{\mathrm{T}}\mathbf{I}/\sigma_i|$) derived from the simulation and experimental results, respectively. The curves of sampling components ($|\boldsymbol{u}_{(i)}^{\mathrm{T}}\mathbf{I}|$) and singular values ($\sigma_i$) are also displayed in the same plot. It can be found that the calculated ($|\mathbf{u}_{(i)}^{\mathrm{T}}\mathbf{I}/\sigma_i|$) does not overall

increase, indicating that the Picard condition is fulfilled and a convergent solution can always be reached.

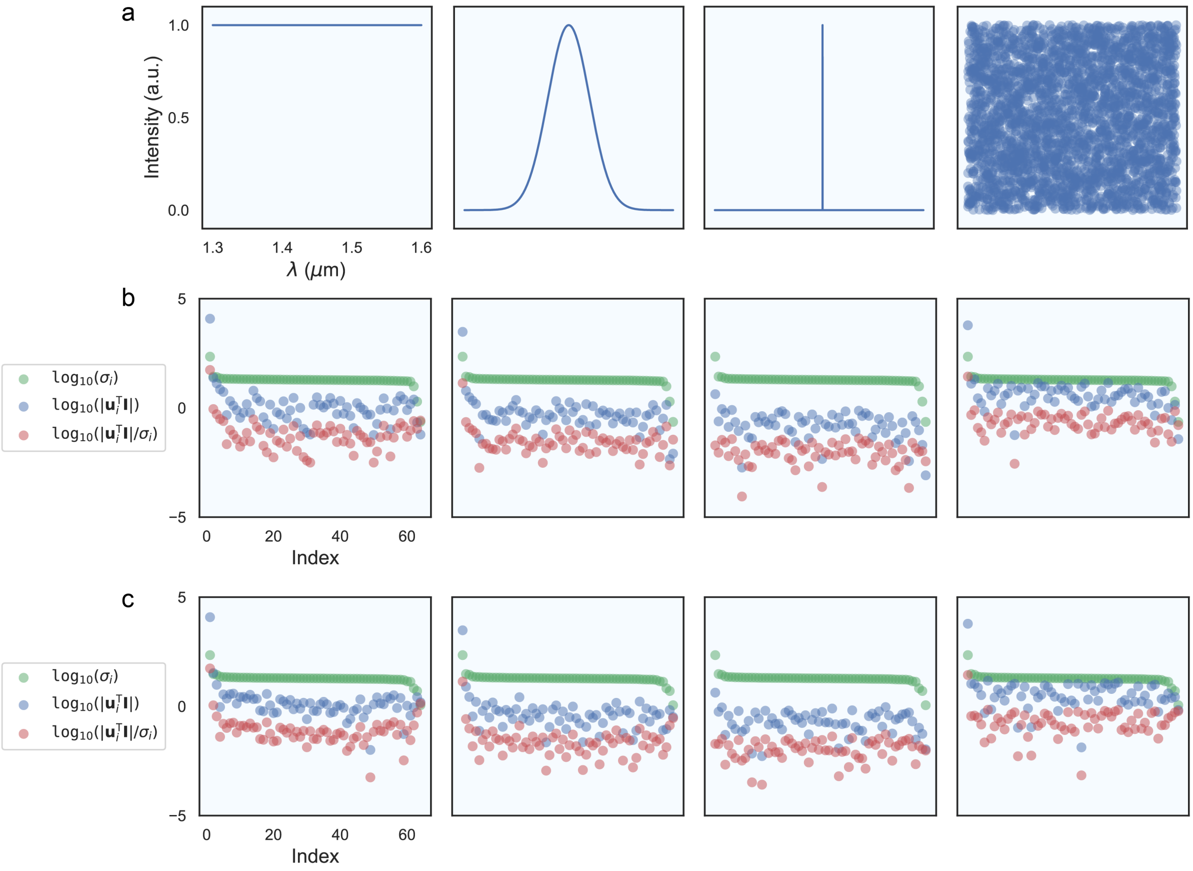

Fig. S10 Picard plots. **a** Four types of testing spectra (**R**). **b, c** Picard plots for the **b** theoretical and **c** experimental transmission matrix.

From another perspective, the priori knowledge of spectral features also helps to accelerate the process. For instance, if we already know that the spectrum solely contains discrete spectral lines, then β can be set as zero and only α needs to be optimized. In other words, α (β) will automatically overwhelm β (α) for narrowband (broadband) spectra during the cross-validation procedure, and the presetting of hyperparameters in the reconstruction of sole narrowband or broadband spectra is only for shortening the optimization time.

Supplementary Note 7

Noise analysis: Much research has been carried out on the theory and modelling of noise in MEMS[S13–16]. The widely recognized paper by Gabrielson examines the impact of mechanical-thermal noise on MEMS and has acted as the foundation for numerous subsequent works[S17]. We examine the thermal and shot noises that influence the designed spectrometer significantly. We present a diagram (**Fig. S11a**) depicting the displacement of the cantilever waveguide caused by thermal noise. By modelling the cantilever as a harmonic oscillator, the mean square displacement $x^2_{\text{thermal noise}}$ is provided by the energy equalization theorem[S18]:

$$\frac{1}{2}k_{\mathrm{B}}T = \frac{1}{2}kx^2_{\text{thermal noise}} \tag{S4}$$

where k_{B} is the Boltzmann constant, T is the absolute temperature, and k is the spring constant of the MEMS cantilever. Thus, the root mean square of $x^2_{\text{thermal noise}}$ is given by:

$$x_{\text{thermal noise}} = \sqrt{\frac{k_{\mathrm{B}}T}{k}} \tag{S5}$$

with $k_{\mathrm{B}} = 1.38\times10^{-23}$ J·K^{-1}, $T = 300$ K, and the spring constant k of the cantilever can be given by:

$$k = \frac{\Delta F}{\Delta x} \tag{S6}$$

where ΔF and Δx denote the variance in the vertical reaction force and the displacement difference between two successive voltage sequences, correspondingly. The reaction force F of the cantilever wave in the vertical direction is simulated in COMSOL Multiphysics, and the spring constant k of the cantilever is determined using displacement data. The alteration in F and k in response to voltage is presented in **Fig. S11b**. Including the incorporation of k into **Eq. S5** enables the calculation of noise displacement caused by thermal noise.

Djuric developed more sophisticated noise models. He combined mechanical-thermal noise with electrical noise sources and presented a formula for determining the shot noise induced by electric charge (**Fig. S11c**)[2]:

$$I_{\text{shot noise}} = \sqrt{2qI_{\mathrm{DC}}B} \tag{S7}$$

where q is the electron charge (1.6×10^{-19} coulombs), I_{DC} is the average DC current (A), and B is the noise bandwidth (Hz).

We conduct a transient analysis of the cantilever with a response time of roughly 12 μs, as illustrated in **Fig. S11d**. To guarantee smooth operation of the device, we have developed a time-variant voltage modulation with interval Δt set at 20 μs. Using COMSOL Multiphysics, the charge Q for a given voltage sequence is simulated for the cantilever, and the average DC current I_{DC} is given by the following equation:

$$I_{\mathrm{DC}} = \frac{\Delta Q}{\Delta t} \tag{S8}$$

where ΔQ denotes the change of Q between two adjacent voltages. The correlation between the change of charge (ΔQ) and direct current (I_{DC}) in relation to voltage is demonstrated in **Fig. S11e**. Here we choose the noise bandwidth as:

$$B = \frac{1}{\Delta t} \tag{S9}$$

The shot noise $I_{\text{shot noise}}$ can be calculated by **Eq. S7**, and the noise charge Q_{noise} caused by the noise current (**Fig. S11f**) can be given by:

$$Q_{\text{noise}} = I_{\text{shot noise}} \cdot \Delta t \tag{S10}$$

For a minuscule cantilever displacement, we can assume a constant capacitance C formed between the cantilever and substrate. Thus, the noise voltage V_{noise} can be calculated using the following equation:

$$C = \frac{Q}{V} = \frac{Q_{\text{noise}}}{V_{\text{noise}}} \tag{S11}$$

thus

$$V_{\text{noise}} = Q_{\text{noise}} \cdot \frac{Q}{V} \tag{S12}$$

Similarly, if the relationship between displacement and voltage remains constant over a limited voltage range, an approximate value for shot noise displacement $x_{\text{shot noise}}$ can be determined:

$$x_{\text{shot noise}} = V_{\text{noise}} \cdot \frac{\Delta x}{\Delta V} \tag{S13}$$

Thermal noise displacement $x_{\text{thermal noise}}$ and shot noise displacement $x_{\text{shot noise}}$ are illustrated in **Fig. 3d** in the main text.

Noise fluctuations cause variations in the optical path difference (OPD), leading to noisy interferograms obtained at the output port. Therefore, denoising is necessary for the spectrometer designed. This analysis examines the impact of two primary types of noise on the displacement of the cantilever beam. It is acknowledged that noise is not limited to these two types. Direct noise removal is extremely challenging. Our denoising model does not directly remove noise, but instead transforms the actual noise issue into a relative noise removal problem, which significantly reduces the difficulty of denoising.

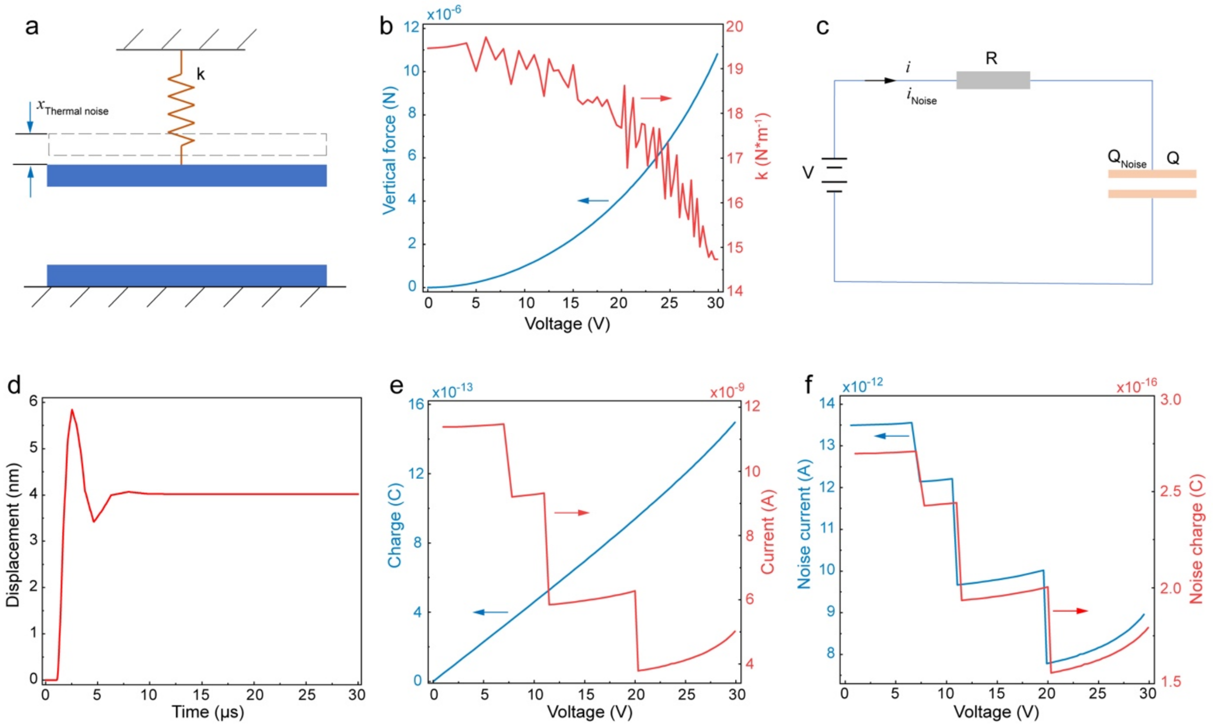

Fig. S11 Noise analysis. **a** Displacement of the cantilever waveguide caused by thermal noise. **b** Reaction force F and spring constant k of the cantilever as functions of voltage. **c** Shot noise model. **d** Time-domain response of the cantilever. **e** Change of charge (ΔQ) and direct current (I_{DC}) as functions of voltage. **f** Noise current ($I_{\text{shot noise}}$) and noise charge (Q_{noise}) under varying voltage.

Supplementary Note 8

Influence of noise on device footprint: The spectral resolution ($\delta\lambda$) for the proposed spectrometer can be given by the Rayleigh criterion[S19]:

$$\delta\lambda = \frac{\lambda^2}{\Delta n_{\mathrm{max}} L} \tag{S14}$$

where Δn_{max} is the maximum Δn during the whole tuning process, which is at the initial state (h = 0 μm), as indicated by **Fig. S1**. At a wavelength of λ = 1.45 μm, the spectral resolution is 42.05 nm according to the Rayleigh criterion. The spectrometer that we have developed has transcended far beyond the resolution specified by the Rayleigh criterion, showing high effectiveness of the MEMS reconfiguration design and the computational reconstruction scheme. As the spectrometer we designed works on the same principle as an FT spectrometer by modulating the OPD (OPD = $L \cdot \Delta n$), the resolution of the spectrometer remains inversely proportional to the OPD[9]. When determining the initial state Δn_0, it is obvious that the OPD is inversely proportional to the length of the waveguide coupler. This means that the resolution is proportional to the length L of the waveguide coupler. Therefore, to enhance the resolution from 1.2 to 0.3 nm at an SNR of 30 dB, the waveguide coupler's length needs to be quadrupled, which is 8120 μm. Similarly, to improve the resolution from 1.2 to 0.4 nm, the length of the waveguide coupler needs to be tripled, which equals 6090 μm. It is apparent that the reduction of noise can significantly decrease the enlarged footprint due to the present of noise and help realize a compact and high-performance spectrometer.

Supplementary Note 9

Noise-oriented autoencoder training: Denoising autoencoder (DAE) aims to learn a representation robust to noise added to the original data. Typically, training a DAE aims to reconstruct the original data with minimal error. However, if the original has a complicated data pattern, training can be time-consuming and may result in underfitting; additionally, if the DAE is trained too specifically on one type of input spectrum, it may lose generalizability to other patterns of input spectra – raising the concern that different input patterns would require different autoencoder settings. Here, we improve the efficiency and performance of DAE by modifying its structure. Instead of reconstructing the input pattern, we reconstruct the noise pattern and then subtract it from the initial input data[S20].

Consider a noisy observation $\mathbf{I}$, which consists of the original data $\mathbf{I}_a$ (referred to as the signal) and the noise $\mathbf{e}$, i.e., $\mathbf{I} = \mathbf{I}_a + \mathbf{e}$. The normal way to train a DAE on signal aims to capture as much information of $\mathbf{I}_a$ as possible, despite $\mathbf{I}$ being a noisy version of the input. As shown in **Fig. S12a**, the parameters of the signal-based DAE model are optimized by minimizing the average reconstruction error in the training phase as follows:

$$\theta_s^*, \theta_s'^* = \arg\min_{\theta,\theta'} \frac{1}{M}\sum_{i=1}^{M} \mathcal{L}\left(\mathbf{I}_a^{(i)}, g_{\theta'}\left(f_\theta\left(\mathbf{I}^{(i)}\right)\right)\right) \tag{S15}$$

where $\mathcal{L}$ is a loss function of MSE between two inputs. Then, the j-th regenerated data $\tilde{\mathbf{I}}_{a,s}^{(j)}$ from $\mathbf{I}^{(j)}$ in the test phase can be obtained as follows for all $j \in \{1, \dots, L\}$:

$$\tilde{\mathbf{I}}_{a,s}^{(j)} = g_{\theta_s'^*}\left(f_{\theta_s^*}\left(\mathbf{I}^{(j)}\right)\right) \tag{S16}$$

This can be understood as an attempt to maximize the lower bound on mutual information $M(\mathbf{I}_a; \mathbf{I})$, or as an attempt to find a manifold where $\mathbf{I}$ represents the data into a low dimensional latent space corresponding to $\mathbf{I}_a$. It may face the problem that the stochastic feature of $\mathbf{I}_a$ to be restored is too complex to regenerate or generalize. Especially in the case of spectrometer, this problem can become evident: if the autoencoder is trained to fit certain input patterns very well, it may have poor generalizability to other input spectra.

In this case, we observe that $\mathbf{e}$ is simpler to regenerate than $\mathbf{I}_a$. $\mathbf{e}$ is not affected by the overall shape of the input spectrum; it is determined by the value of each data point or its limited neighbour data points in the spectrum. Thus, we naturally come up with the intuition that training the DAE by learning $\mathbf{e}$ and subtracting it from $\mathbf{I}$, which should be more effective than learning $\mathbf{I}_a$ directly. The training and testing phases of training on noise are depicted in **Fig. S12b**. The parameters of the noise-based DAE can be optimized as follows:

$$\theta_n^*, \theta_n'^* = \arg\min_{\theta,\theta'} \frac{1}{M}\sum_{i=1}^{M} \mathcal{L}\left(\mathbf{e}^{(i)}, g_{\theta'}\left(f_\theta\left(\mathbf{I}^{(i)}\right)\right)\right) \tag{S17}$$

Notice that the only difference from (1) is that $\mathbf{I}_a^{(i)}$ is replaced by $\mathbf{e}^{(i)}$. During training phase, the $\mathbf{e}^{(i)}$ is derived by subtracting the ground truth from the input sample $\mathbf{I}^{(i)}$. In testing phase, the ground truth is no longer needed by employing the trained DAE to predict $\mathbf{e}^{(i)}$. Let $\tilde{\mathbf{I}}_{a,n}^{(j)}$ denote the j-th regenerated data, which can be represented as follows for all $j \in \{1, \dots, L\}$:

$$\tilde{\mathbf{I}}_{a,n}^{(j)} = \mathbf{I}^{(j)} - g_{\theta_n'^*}\left(f_{\theta_n^*}(\mathbf{I}^{(j)})\right) \tag{S18}$$

To verify that the noise-based DAE improves the efficiency of denoising interferograms, we conducted verification through two aspects:

1) When the DAE is trained on a certain type of input spectrum, the noise-based DAE exhibits stronger generalizability than the signal-based DAE. A training set of 600,000 samples of two-peak interferograms with 30 dB SNR is used. As shown in **Fig. S12c**, the MSE error between each pair of ground truth and predicted output is depicted. The blue colour represents the signal-based results (MSE between the pair $(\tilde{\mathbf{I}}_{a,s}^{(j)}, \mathbf{I}_a^{(j)})$). The purple colour represents the noise-based results (MSE between the pair $(\tilde{\mathbf{I}}_{a,n}^{(j)}, \mathbf{I}_a^{(j)})$). Since the model is trained specifically on two-peak data, both DAEs show great performance and are very close. However, when the model is extended to unseen data in the training set, such as three-peak and four-peak data, although both models show generalizability, the noise-based model is superior, with a lower level of MSE. The reconstructed interferograms and the reconstructed spectra are shown in **Fig. S12d**, in which the distinct areas where the noise-based DAE predicts more accurately are circled in red. Cases are depicted where the signal-based DAE fails to reconstruct the spectrum, while the noise-based DAE successfully reconstructs the spectra thanks to the improved accuracy in predicting the input. The model is further extended to various noise levels, and we observe that the performance superiority and generalizability of the noise-based model persist, as shown in **Fig. S12e**.
2) By training on a mixture of various input spectrum types, we avoid overfitting to a certain type (like the two-peak in aspect 1), and significantly reducing the required data volume. A mixed dataset of two-peak, three-peak, and four-peak data is used, with 10,000 samples for each type (representing a 10× decrease in data volume and training time). Since the model now will not be overfit to a certain type of input spectrum, but rather focus on learning the noise pattern, the MSE is further decreased. As shown in **Fig. S12f,** compared to training on two-peak data only, the MSE error is decreased to a level below 0.001 for all data included in the training set. This method can be extended to other input spectra with high generalizability. For example, on a testing five-peak dataset, the MSE is 0.0009, and on a testing broadband spectrum, the MSE is 0.0014, with almost precisely reconstructed

input data (**Fig. S12g,** the reconstructed spectra are also shown). A more diverse dataset is always preferred, not because we need to see the input spectrum shape to reconstruct it, but to prevent the network from overfitting to one type of input data. **Fig. S12h** shows the generalizability to multiple noise levels. If the SNR level difference exceeds 10 dB, generalizability will decrease. However, in practical situations, it is acceptable to test the approximate noise level and select the appropriate model.

With these two demonstrations, we showcase the capability of our noise-based autoencoder scheme to adapt to unseen input waveforms without the need for retraining for each specific kind of pattern.

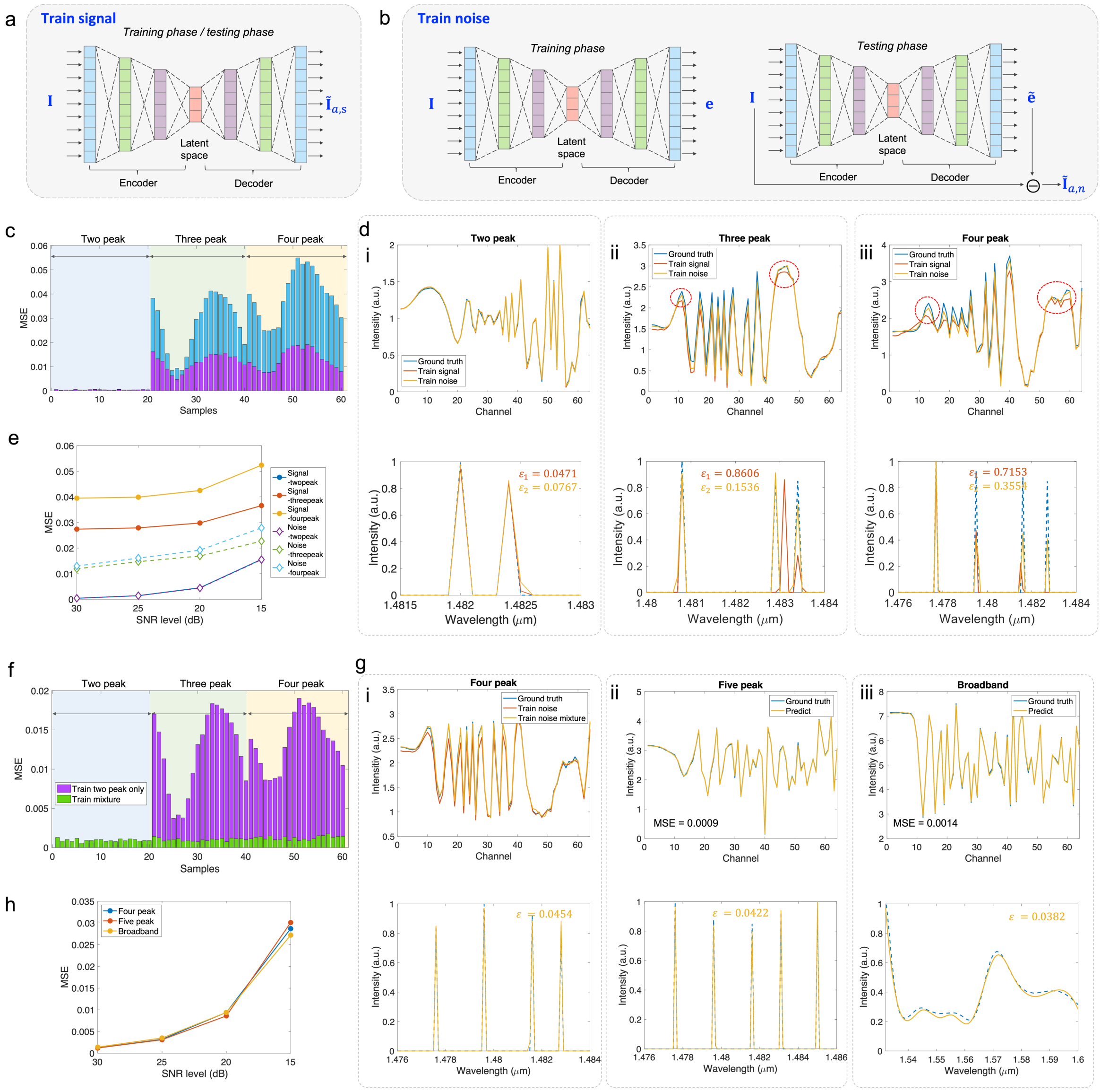

Fig. S12 Noise-oriented training of the denoising autoencoder. **a** Architecture of signal-oriented training/testing. **b** Architecture of noise-oriented training/testing. **c** MSE comparison between signal- and noise-oriented training schemes when training on two-peak data only, and extending

to three-peak and four-peak data to evaluate generalizability. **d** Predicted output of the denoising autoencoder and the reconstructed spectrum. **e** Generalizability across multiple SNR levels. **f** MSE comparison of training on one type of data only versus training on a mixed dataset. A mixed dataset with diverse input patterns is preferred as it prevents the model from overfitting to a single input signal pattern and encourages learning the noise pattern. **g** Predicted output of the denoising autoencoder and the reconstructed spectrum, which show great generalizability to unseen data in the training set, including five-peak and broadband datasets, with an MSE level of 0.001, allowing for nearly lossless reconstruction of the input spectrum. **h** Generalizability to multiple SNR levels.

Supplementary Note 10

CAED at lower SNR levels: Considering that some real-life applications require working in low SNR environments, we have also investigated the denoising performance of our CAED mechanism at lower SNR levels of 20, 15, and 8 dB, as shown in **Fig. S13**. Both the reconstruction resolutions with and without denoising worsen with increasing noise level (i.e., decreasing SNR). However, for all the SNR levels, the denoising by CAED significantly improves the reconstruction resolution (for 20 dB SNR, from 1.5 to 0.5 nm; for 15 dB SNR, from 1.7 to 0.6 nm; for 8 dB SNR, from 2.1 to 0.8 nm). Additionally, as mentioned in the main text, better denoising results are expected to be achieved if we train the autoencoder using a noise dataset corresponding to these lower SNR values. Therefore, our CAED mechanism would be effective across the whole SNR range in real-life applications.

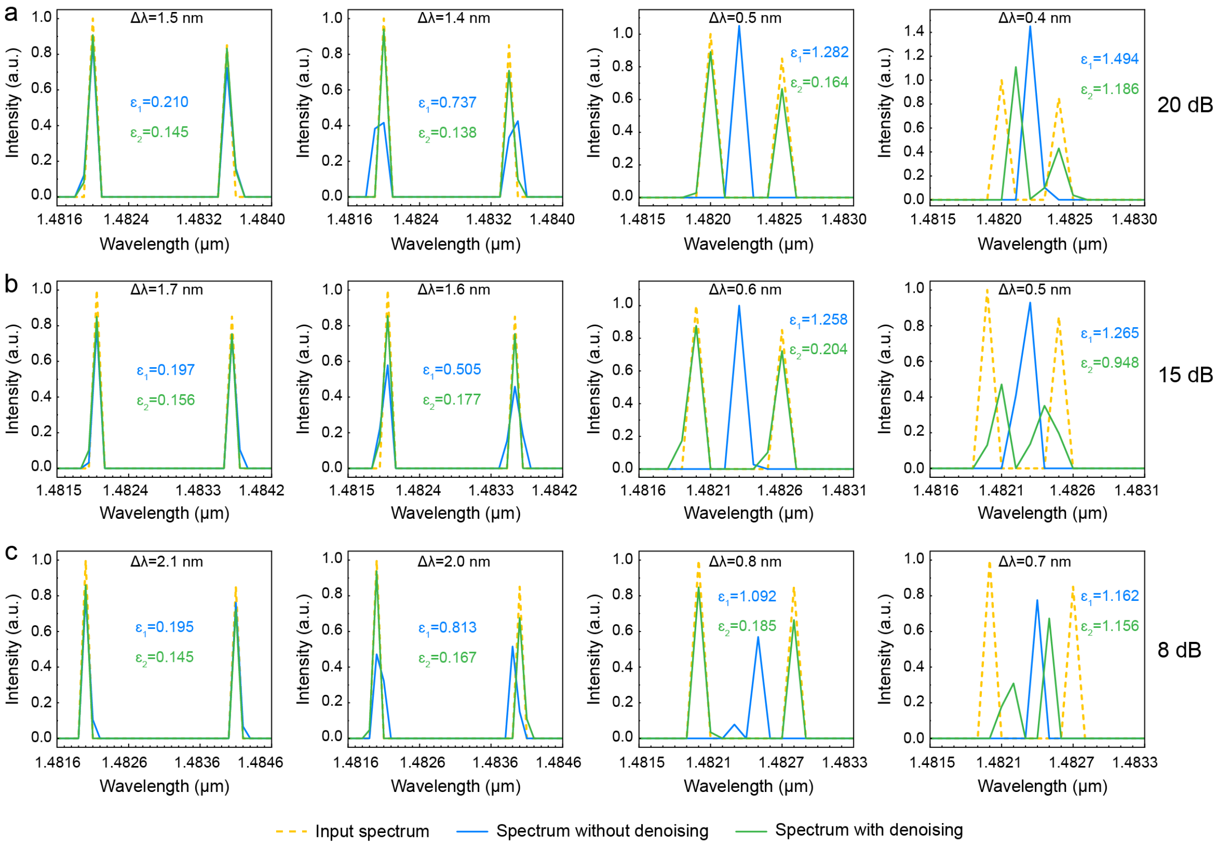

Fig. S13 Dual-wavelength spectrum reconstruction under lower SNR levels.

Supplementary Note 11

Effectiveness of CAED exceeds dataset mapping: In autoencoder training purposes, the dual-wavelength interferograms used to comprise our noise dataset have wavelength spacings of less than 10 nm. To confirm whether the autoencoder is capable of reducing noise without causing reconstruction distortion when the wavelength spacings of the dual-wavelength interferograms exceed 10 nm, we have selected three dual-wavelength interferograms with wavelength spacings of 20, 30, and 50 nm, respectively. The reconstruction results are displayed in **Fig. S14**. In all three cases, the two wavelengths are accurately retrieved, and the intensity reconstruction accuracy remains consistent even with increasing wavelength spacings. This demonstrates that the wavelength spacings of less than 10 nm in the noise dataset do not impact generalizability to other spacings.

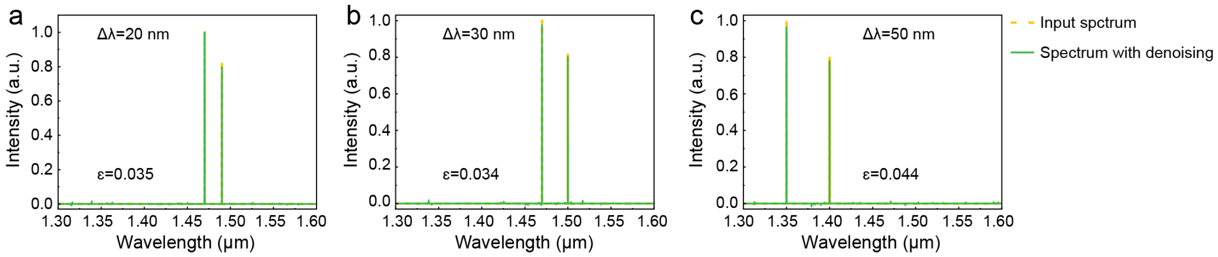

Fig. S14 Reconstruction of dual-wavelength spectrum with different wavelength spacings of **a** 20 nm, **b** 30 nm, **c** 50 nm.

Supplementary Note 12

Robustness to temperature fluctuations: As stated in Ref. [S21], for computational spectrometers using broadband filters with non-zero transmissions within a wide optical range, the temperature change shifts the transmission spectrum, but for the optical range of interest, the transmissions are still non-zero and the spectral information would not be lost. Temperature change will only generate a new sampling matrix, which could be recorded at the calibration stage, so that the input spectra can still be resolved at different temperatures, simply by choosing the correct calibration matrix. Therefore, employing a simple and cheap temperature sensor is adequate for computational spectrometers to work in a broad temperature range.

The above statement is also true for our MEMS spectrometer, because the MEMS-tunable directional coupler at each tuning state can be regarded as a broadband filter as well and will not lose spectral information under temperature change. Therefore, input spectra can be successfully reconstructed without performance degradation, as long as the calibration matrix at each temperature is pre-recorded. In order to verify this statement, we have also tested the spectral reconstruction performance of our MEMS spectrometer at fluctuating temperatures in the range of 10-70 °C, which is consistent with Ref. [S21] and covers the reasonable operating temperature range for practical applications. The calibration matrices at these three temperatures are first collected and then utilized to reconstruct dual-wavelength spectra at the corresponding temperatures. As shown in **Fig. S15**, both the MEMS spectrometer and the CAED mechanism work well at all these three temperatures, thus maintaining a denoised resolution of 0.4 nm across the whole operating temperature range.

Another aspect of the temperature tolerance is the temperature fluctuation range in which the input signal can still be recovered to the accuracy of the spectrometer resolution without the recollection of the calibration matrix[S8,22]. To estimate this aspect of temperature tolerance, we simulate the interferogram of a single-wavelength spectrum with a randomly selected wavelength (λ = 1532 nm) at different temperatures by changing the refractive index of the Si according to the thermo-optic coefficient, $\mathrm{d}n/\mathrm{d}T \approx 1.8\times10^{-4}\ \mathrm{K}^{-1}$ [S23]. We then use the calibration matrix obtained with no temperature variation to perform the spectrum reconstructions. As shown in **Fig. S16**, with a temperature variation of ±8 °C, the input signal can still be recovered to the accuracy of the spectrometer resolution, i.e., with the offset of center wavelength remaining within ±0.4 nm.

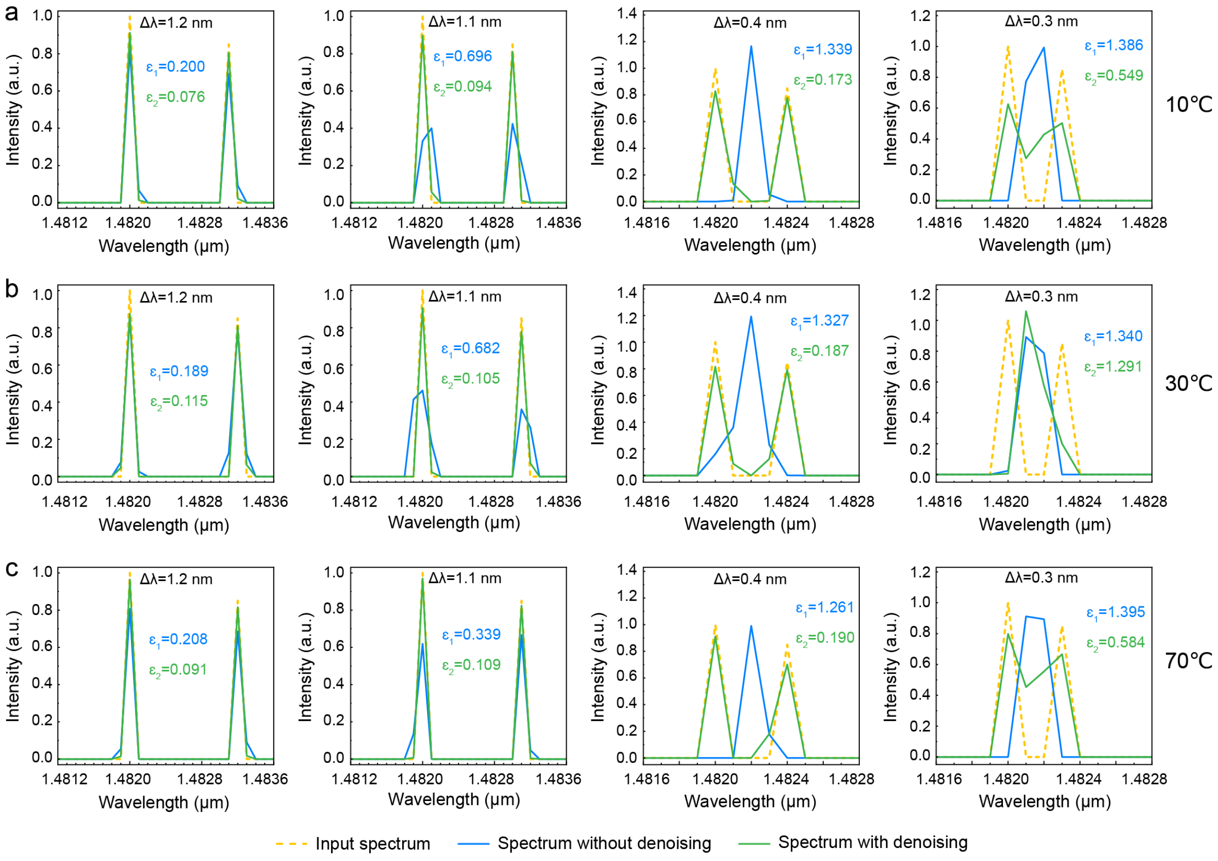

Fig. S15 Dual-wavelength spectrum reconstruction at different temperatures with recollection of the calibration matrix.

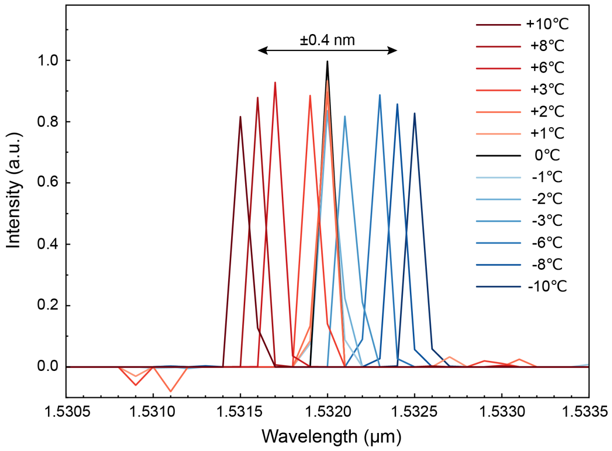

Fig. S16 Single-wavelength spectrum reconstruction at different temperatures without recollection of the calibration matrix.

Supplementary Note 13

Tolerance to fabrication errors: The deviation of waveguide widths (δW_{WG}) is the primary source of fabrication defects, which will lead to differences in the calibration matrix. Nonetheless, since the calibration matrix of each device will be collected prior to its use for spectrum reconstruction, such differences among devices may be inconsequential so long as some key figures of merit remain with marginal fluctuations. There are two crucial features in the spectrum reconstruction, i.e., the ability to identify fine spectral details and the decorrelation among all wavelength channels, which can be quantified by the self-correlation function [$C(\Delta\lambda)$] and singular values (σ_i) of the calibration matrix[S10]. We compare the $C(\Delta\lambda)$ and σ_i between the spectrometers with or without δW_{WG} of ±20 nm, which is attainable for most photonic foundries. As depicted in **Fig. S17**, the self-correlation width shows deviation within one wavelength step of 0.1 nm, and σ_i also does not show significant change, indicating satisfactory robustness against fabrication defects.

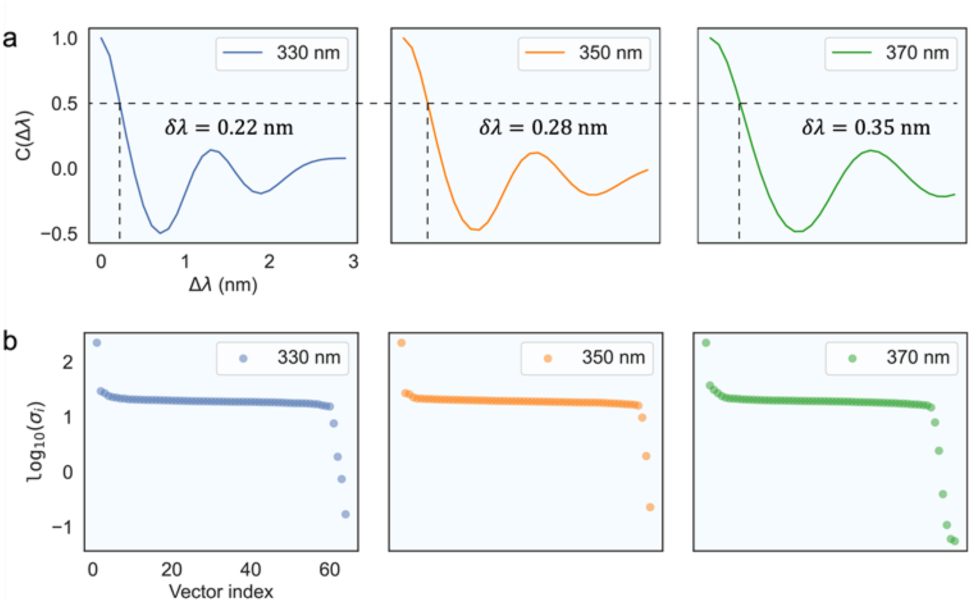

Fig. S17 Tolerance analysis of fabrication errors. Calculated **a** self-correlation functions [$C(\Delta\lambda)$] and **b** singular values (σ_i) of the calibration matrices with waveguide width deviations δW_{WG} of ±20 nm.

Supplementary Note 14

Reconstruction of a more broadband spectrum: The reconstruction of a continuous spectrum spanning the entire 300 nm bandwidth from 1.3 to 1.6 μm is simulated. White noise corresponding to 30 dB SNR is added to the simulation. As shown in **Fig. S18**, our CAED-facilitated MEMS spectrometer is able to accurately reconstruct the spectrum with a low relative error ε of 0.046, providing additional evidence of the 300 nm bandwidth.

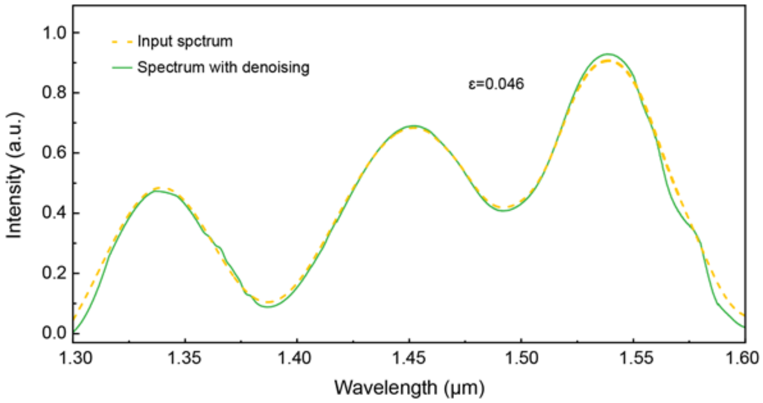

Fig. S18 Simulated reconstruction of a continuous spectrum spanning from 1.3 to 1.6 μm.

Supplementary Note 15

Comparison of reported on-chip spectrometers:

Table S2. Performance comparison of reported on-chip spectrometers.

Type	Structure	Resolution (nm)	Bandwidth (nm)	Footprint (μm^2)	Voltage (V)	Power consumption (mW)
DO[S24]	Disordered structure	3.4	40	50×200	/	/
DO[S25]	Disordered structure	0.25	30	12.8×30	/	/
DO[S22]	Disordered structure	0.75	25	50×100	/	/
DO[S26]	PhC reflector	2.5	55	480×800	/	/
DO[S27]	Branched waveguide	3	100	12×63	/	/
DO[S28]	Echelle grating	1.2	30	1100×1420	/	/
NF[S29]	FP cavity array	0.51	102.7	43×600	8.5	873
NF[S9]	Two coupled MRRs	0.04	100	60×60	NM	85
NF[S30]	Euler MRR + cascaded MRR array	0.005	10	3.5×10^5	NM	504
NF[S31]	MRR array	0.6	60	1×10^6	/	/
NF[S32]	MRR + random gratings	0.2	60	215×310	NM	33.23
FT[S33]	Single MZI	3.05	56.18	1×10^6	200	5100
FT[S34]	SWIFTS	4	96	22×512	/	/
FT[S11]	tFTS + SHS	0.125	200	5500×6000	135	2400
FT[S35]	Cascaded DCs	4	300	NM	/	/
FT[S36]	MRR + MZI	0.47	90	NM	NM	1835
FT[S19]	MZI with embedded switches	0.2	20	630×2820	NM	99
CR[S7]	Stratified waveguide filter array	0.45	180	35×260	/	/
CR[S37]	PhC nanobeam cavity array	5	70	6×111	/	/
CR[S38]	Cascaded PhC nanobeam cavities	0.32	16	18×250	NM	90

CR[S8]	Cascaded MZIs	0.01	200	1900×3700	NM	2100
CR[S39]	Distributed MRRs	0.03	115	2000×7600	/	/
**CR (This work)**	**Single DC**	**0.4 (with denoising) 1.2 (without denoising)**	**300**	**38×2030**	**29.9**	**6.987×10^{-2}**

DO: dispersive optics
NF: narrowband filtering
FT: Fourier transform
CR: computational reconstruction
PhC: photonic crystal
FP: Fabry-Pérot
MRR: microring resonator
MZI: Mach-Zehnder interferometer
SWIFTS: stationary-wave integrated Fourier-transform spectrometer
tFTS: tunable Fourier-transform spectrometer
SHS: spatial heterodyne spectrometer
DC: directional coupler
NM: not mentioned

Supplementary Note 16

Multi-stage structure of MEMS spectrometer: Considering that our proposed MEMS spectrometer is just in its nascent stage of development as a physically single-stage and single-channel device (similar to the Michelson interferometer in the early days of on-chip spectrometers), it holds immense potential for the development of multiple physical stages or channels. On that note, some notable works proposing strategies to enhance sampling channel diversity offer valuable insights for the further development of MEMS spectrometers[S8,39]. These strategies are indeed applicable to the expansion of the single-channel MEMS spectrometer. Here, we discuss through simulations how the self-/cross-correlations and reconstruction resolution of MEMS spectrometers can be improved by employing multi-stage structures.

Inspired by the multi-stage design demonstrated in Ref. [S8], here we investigate a multi-stage structure in improving the performance of the MEMS spectrometer through simulation. We assessed multiple cascaded MEMS spectrometers, examining m stages. We explore every conceivable combination of voltage modulation channels, totalling n^m channels, as shown in **Fig. 7a**. To accommodate computational limitations, we choose $n = 8$ and focus on scenarios where m equals 2 and 3. The computed self- and cross-correlations are compared to those of the single-stage device (the one in the main text with 64 channels), as depicted in **Figs. S19a, b**. As the number of stages and sampling channels increases, the decreased self-correlation width $\delta\lambda$ indicates improved spectral reconstruction resolution. The improvement is also reflected in the minimum value of the self-correlation function, which represents a measure of anti-correlation and is preferred to be close to zero. Additionally, the resolved resolution is expected to significantly exceed the value of $\delta\lambda$ because of the exponentially increased number of sampling channels, as suggested by Ref. [S8]. To verify this, we further conduct the dual-wavelength spectra reconstruction using the calibration matrix of the 3-stage spectrometer (shown in **Fig. 7b** and also **Fig. S19c**). **Figure S19d** plots the transmission spectra of several representative sampling channels, illustrating a high degree of spectral randomness and sufficient decorrelation between sampling channels. The dual-wavelength spectra reconstruction results as depicted in **Fig. S19e** show a high noise-free resolution of 15 pm.

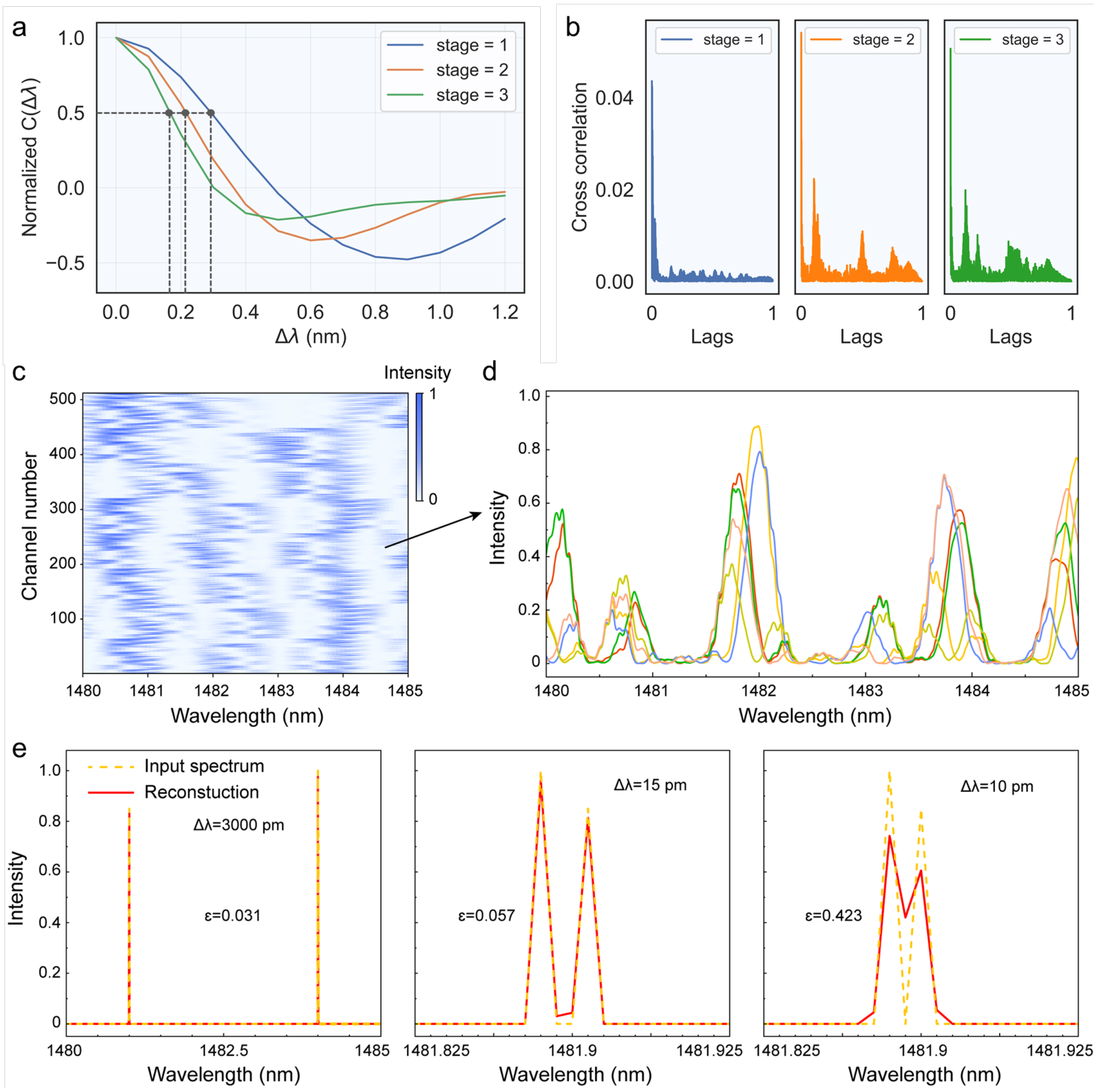

Fig. S19 Performance of multi-stage MEMS spectrometer. **a** Self- and **b** Cross-correlations varying with the stage number. **c** Calibration matrix of the 3-stage spectrometer. **d** Several example channel spectral responses, featuring significant spectral differences. **e** Dual-wavelength spectra reconstruction, showing a noise-free resolution of 15 pm.

We further investigate the performance of this multi-stage MEMS spectrometer under a noisy environment. The SNR of this multi-stage device is estimated to be 19 dB, following the noise analysis method described in **Supplementary Note 7**. The higher noise level is due to the cascaded structure and longer total coupling length. Next, white noise according to the 19 dB SNR is added to the interferograms, which are then used for the spectrum reconstructions. As shown by the dual-wavelength spectrum reconstruction results in **Figs. 7c-f**, our current denoising autoencoder improves the reconstruction resolution from 90 to 40 pm, approaching

the noise-free value of 15 pm. The denoising performance is expected to be further enhanced by accordingly optimizing the design of the autoencoder network. We further conduct the reconstructions of diverse input spectra, including triple-wavelength, broadband, and mixed broadband/narrowband spectra. As shown in **Figs. S20a-c**, all these spectra can be reconstructed with a low relative error ε.

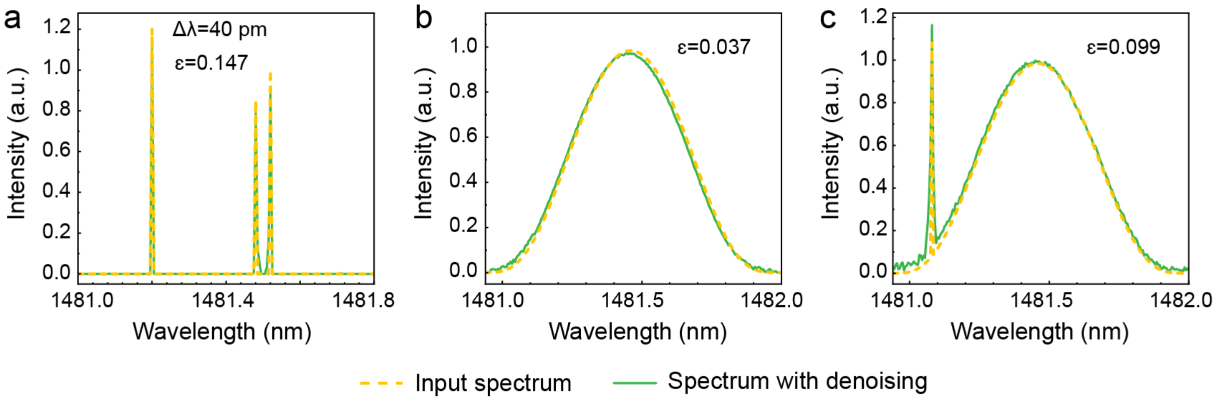

Fig. S20 Performance of the multi-stage MEMS spectrometer under 19 dB SNR. **a-c** Reconstruction of a diverse range of incident spectra: **a** triple-wavelength spectrum, **b** broadband spectrum, **c** mixed broadband/narrowband spectrum.

The above results demonstrate the benefits of employing a multi-stage structure, with further enhancements achievable through an increase in channels. This implies large room for further in-depth research about specific network/cascaded structures for enhancing MEMS spectrometers, which will raise broad research interest and thus lead to further advancements.

Supplementary Note 17

In-plane configuration: The footprint of our device can be further reduced by reducing the coupling gap of the waveguide coupler. A smaller coupling gap will lead to stronger coupling between the cantilever and straight waveguides and thus a larger Δn, thus leading to an improved resolution, as suggested by **Eq. S14**. The coupling gap can be further narrowed by using more advanced lithography. To be more practical, here we propose a new spectrometer design, in which the cantilever actuator is replaced with a comb-drive actuator, thus changing the current out-of-plane reconfiguration scheme to an in-plane reconfiguration scheme, as illustrated in **Fig. S21a**. The waveguides are kept with the same thickness of 220 nm and width of 350 nm. The two waveguides are initially separated by 460 nm. The comb fingers in the comb-drive actuator measure 250 nm in width and 4 μm in length. Upon the application of bias voltage between the fixed and movable fingers, the movable waveguide is pushed toward the fixed waveguide, reducing the coupling gap. As shown in **Fig. S21b**, under an applied bias voltage of 33.6 V, the coupling gap can be reduced to 50 nm, a value beyond the resolution offered by common silicon photonics foundries, leading to a drastic increase of Δn. As a result, a noise-free reconstruction resolution of 0.3 nm can be achieved with a coupling length of only 500 μm using the calibration matrix shown in **Fig. S21c**, as depicted in **Fig. S21d**.

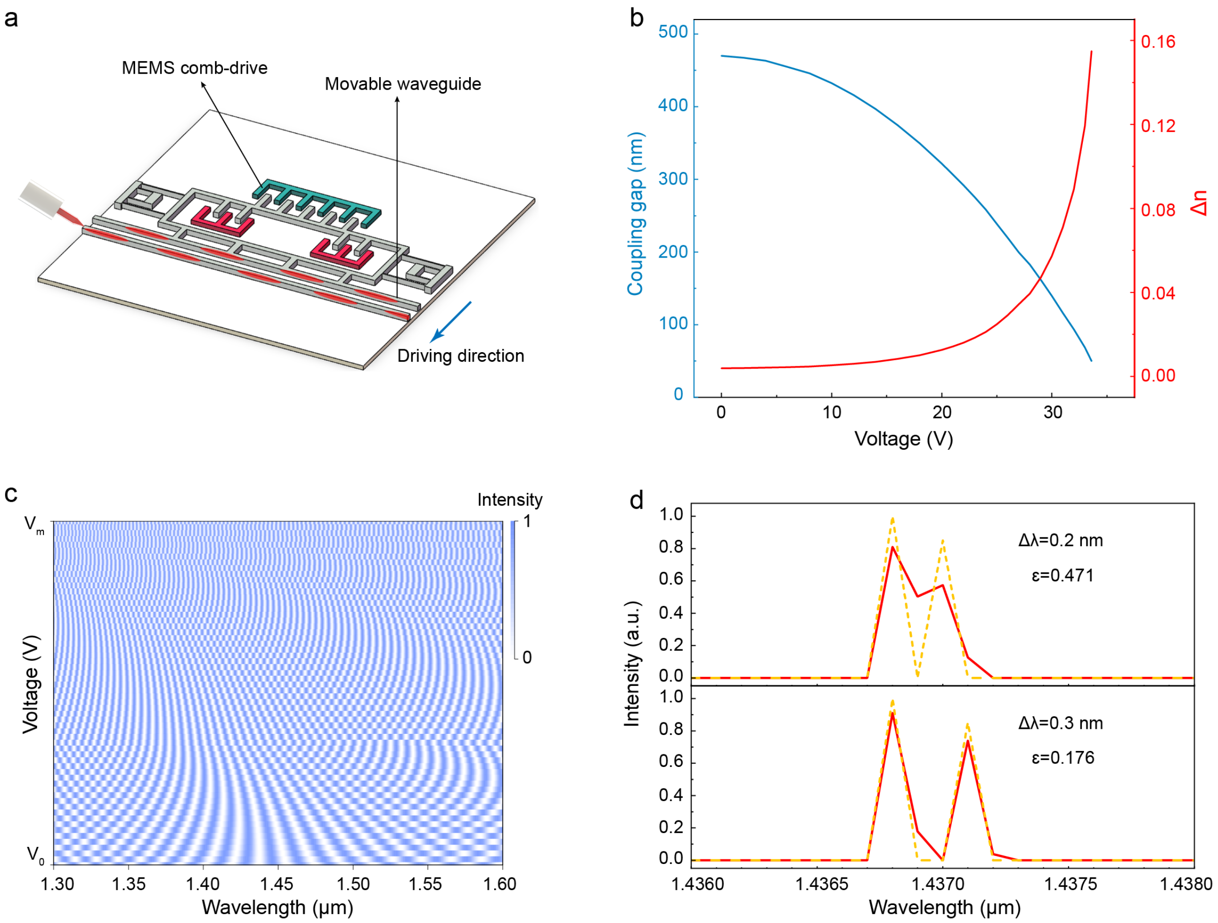

Fig. S21 Design of a compact MEMS spectrometer by employing in-plane reconfiguration. **a** Schematic illustration of the spectrometer, where the in-plane reconfiguration is realized by the integrated pair of comb-drive actuators. **b** Coupling gap and Δn as functions of applied bias voltage. **c** Calibration matrix of the proposed spectrometer. **d** Dual-wavelength spectrum reconstruction showing a resolution of 0.3 nm.

Supplementary Note 18

Discussion on driving voltage: As can be seen from **Table S2**, the driving voltage of our device is not high if compared with the reported works using thermo-optic tuning[S11,33]. Nonetheless, the driving voltage of our device is still out of reach of CMOS electronics which typically provide a driving voltage of several volts. In order to make our device able to be driven by CMOS electronics in practical applications, we can make improvements in two aspects:

1) The driving voltage provided by CMOS electronics can be amplified to the required value by a high-voltage amplifier and then delivered to our device. High-voltage amplifiers have been widely adopted to drive various MEMS actuators, including electrostatic parallel-plate actuator[S40], electrostatic comb-drive actuator[S41], piezoelectric actuator[S42], etc.
2) The cantilever can be designed softer to lower the driving voltage to several volts by lengthening the cantilever, thinning the Al layer, and enlarging the releasing holes on the cantilever, as shown in **Fig. S22**. It is worth noting that a trade-off between driving voltage and mechanical robustness as well as response time needs to be made.

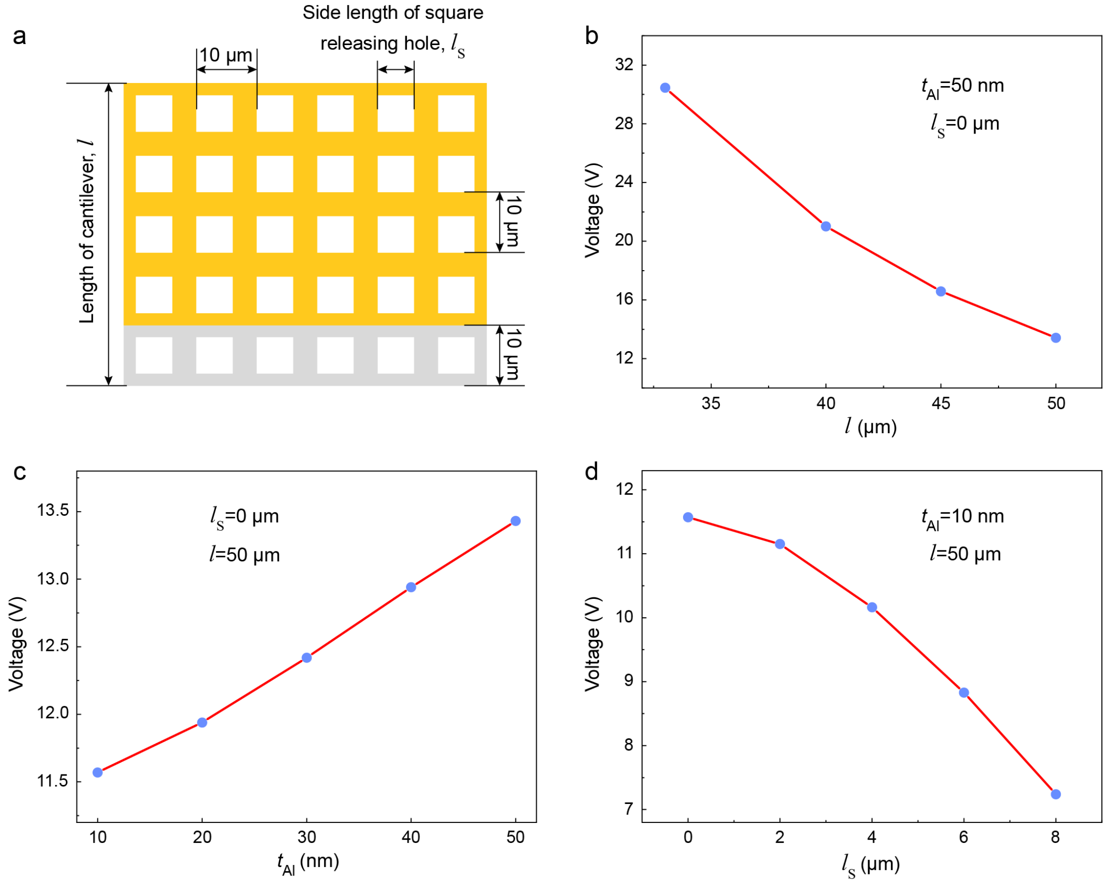

Fig. S22 Driving voltage. **a** Top-view schematic illustration of the MEMS cantilever. **b-d** Driving voltage required to achieve 0.7 μm displacement with varying **b** length of the cantilever, **c** thickness of the Al layer, **d** side length of the square releasing holes.

References

S1. O'Brien, G., Monk, D. J. & Lin, L. MEMS cantilever beam electrostatic pull-in model. *Proc. SPIE* **4593**, 31–41 (2001).

S2. Qiao, Q. *et al.* MEMS-Enabled On-Chip Computational Mid-Infrared Spectrometer Using Silicon Photonics. *ACS Photonics* **9**, 2367–2377 (2022).

S3. Cheben, P., Halir, R., Schmid, J. H., Atwater, H. A. & Smith, D. R. Subwavelength integrated photonics. *Nature* **560**, 565–572 (2018).

S4. Chen, L. R., Member, S. & Wang, J. Subwavelength Grating Waveguide Devices for Telecommunications Applications. *IEEE J. Sel. Top. Quantum Electron.* **25**, 8200111 (2019).

S5. Fukazawa, T., Hirano, T., Ohno, F. & Baba, T. Low Loss Intersection of Si Photonic Wire Waveguides. *Jpn. J. Appl. Phys.* **43**, 646–647 (2004).

S6. Gyger, S. *et al.* Reconfigurable photonics with on-chip single-photon detectors. *Nat. Commun.* **12**, 1408 (2021).

S7. Li, A. & Fainman, Y. On-chip spectrometers using stratified waveguide filters. *Nat. Commun.* **12**, 2704 (2021).

S8. Yao, C. *et al.* Integrated reconstructive spectrometer with programmable photonic circuits. *Nat. Commun.* **14**, 6376 (2023).

S9. Xu, H., Qin, Y., Hu, G. & Tsang, H. K. Breaking the resolution-bandwidth limit of chip-scale spectrometry by harnessing a dispersion-engineered photonic molecule. *Light Sci. Appl.* **12**, 64 (2023).

S10. Xu, H., Qin, Y., Hu, G. & Tsang, H. K. Cavity-enhanced scalable integrated temporal random-speckle spectrometry. *Optica* **10**, 1177–1188 (2023).

S11. Xu, H., Qin, Y., Hu, G. & Tsang, H. K. Scalable integrated two-dimensional Fourier-transform spectrometry. *Nat. Commun.* **15**, 436 (2024).

S12. Hansen, P. C. The discrete picard condition for discrete ill-posed problems. *BIT* **30**, 658–672 (1990).

S13. Mohd-Yasin, F., Nagel, D. J. & Korman, C. E. Noise in MEMS. *Meas. Sci. Technol.* **21**, 012001 (2010).

S14. Djurić, Z. Mechanisms of noise sources in microelectromechanical systems. *Microelectron. Reliab.* **40**, 919–932 (2000).

S15. Behera, M. *et al.* Accurate Simulation of RF MEMS VCO performance including phase noise. *J. Microelectromechanical Syst.* **14**, 313–325 (2005).

S16. Rocha, L. A., Cretu, E. & Wolffenbuttel, R. F. Measuring and interpreting the mechanical–thermal noise spectrum in a MEMS. *J. Micromechanics Microengineering* **15**, S30–S38 (2005).

S17. Gabrielson, T. B. Mechanical-Thermal Noise in Micromachined Acoustic and Vibration sensors. *IEEE Trans. Electron Devices* **40**, 903–909 (1993).

S18. Krause, A. G., Winger, M., Blasius, T. D., Lin, Q. & Painter, O. A high-resolution microchip optomechanical accelerometer. *Nat. Photonics* **6**, 768–772 (2012).

S19. Kita, D. M. *et al.* High-performance and scalable on-chip digital Fourier transform spectroscopy. *Nat. Commun.* **9**, 4405 (2018).

S20. Lee, W. H., Ozger, M., Challita, U. & Sung, K. W. Noise Learning-Based Denoising Autoencoder. *IEEE Commun. Lett.* **25**, 2983–2987 (2021).

S21. Li, A. *et al.* An integrated single-shot spectrometer with large bandwidth-resolution ratio and wide operation temperature range. *PhotoniX* **4**, 29 (2023).

S22. Redding, B., Liew, S. F., Sarma, R. & Cao, H. Compact spectrometer based on a disordered photonic chip. *Nat. Photonics* **7**, 746–751 (2013).

S23. Komma, J., Schwarz, C., Hofmann, G., Heinert, D. & Nawrodt, R. Thermo-optic coefficient of silicon at 1550 nm and cryogenic temperatures. *Appl. Phys. Lett.* **101**, 041905 (2012).

S24. Hartmann, W. *et al.* Waveguide-Integrated Broadband Spectrometer Based on Tailored Disorder. *Adv. Opt. Mater.* **8**, 1901602 (2020).

S25. Hadibrata, W., Noh, H., Wei, H., Krishnaswamy, S. & Aydin, K. Compact, High-resolution Inverse-Designed On-Chip Spectrometer Based on Tailored Disorder Modes. *Laser Photon. Rev.* **15**, 2000556 (2021).

S26. Momeni, B., Askari, M., Shah Hosseini, E., Atabaki, A. & Adibi, A. An on-chip silicon grating spectrometer using a photonic crystal reflector. *J. Opt.* **12**, 035501 (2010).

S27. Zheng, Z., Zhu, S., Chen, Y., Chen, H. & Chen, J. Towards integrated mode-division demultiplexing spectrometer by deep learning. *Opto-Electronic Sci.* **1**, 220012 (2022).

S28. Xie, S., Meng, Y., Bland-Hawthorn, J., Veilleux, S. & Dagenais, M. Silicon nitride/silicon dioxide echelle grating spectrometer for operation near 1.55 μm. *IEEE Photonics J.* **10**, 4502207 (2018).

S29. Sun, C. *et al.* Broadband and High-Resolution Integrated Spectrometer Based on a Tunable FSR-Free Optical Filter Array. *ACS Photonics* **9**, 2973–2980 (2022).

S30. Zhang, L. *et al.* Ultrahigh-resolution on-chip spectrometer with silicon photonic resonators. *Opto-Electronic Adv.* **5**, 210100 (2022).

S31. Xia, Z. *et al.* High resolution on-chip spectroscopy based on miniaturized microdonut resonators. *Opt. Express* **19**, 12356–12364 (2011).
S32. Sun, C. *et al.* Integrated Microring Spectrometer with In-Hardware Compressed Sensing to Break the Resolution-Bandwidth Limit for General Continuous Spectrum Analysis. *Laser Photon. Rev.* **17**, 2300291 (2023).
S33. Souza, M. C. M. M., Grieco, A., Frateschi, N. C. & Fainman, Y. Fourier transform spectrometer on silicon with thermo-optic non-linearity and dispersion correction. *Nat. Commun.* **9**, 665 (2018).
S34. le Coarer, E. *et al.* Wavelength-scale stationary-wave integrated Fourier-transform spectrometry. *Nat. Photonics* **1**, 473–478 (2007).
S35. Li, L. *et al.* Design of an on-chip Fourier transform spectrometer using waveguide directional couplers and NEMS. *Opt. Express* **26**, 30362–30370 (2018).
S36. Zheng, S. N. *et al.* Microring resonator-assisted Fourier transform spectrometer with enhanced resolution and large bandwidth in single chip solution. *Nat. Commun.* **10**, 2349 (2019).
S37. Cheng, Z. *et al.* Generalized Modular Spectrometers Combining a Compact Nanobeam Microcavity and Computational Reconstruction. *ACS Photonics* **9**, 74–81 (2022).
S38. Zhang, J., Cheng, Z., Dong, J. & Zhang, X. Cascaded nanobeam spectrometer with high resolution and scalability. *Optica* **9**, 517–521 (2022).
S39. Yao, C. *et al.* Broadband picometer-scale resolution on-chip spectrometer with reconfigurable photonics. *Light Sci. Appl.* **12**, 156 (2023).
S40. Horenstein, M. N. *et al.* Ultra-low-power multiplexed electronic driver for high resolution deformable mirror systems. *Proc. SPIE* **7930**, 79300M (2011).
S41. Takahashi, K. *et al.* Monolithic integration of high voltage driver circuits and MEMS actuators by ASIC-like postprocess. in *The 13th International Conference on Solid-State Sensors, Actuators and Microsystems (TRANSDUCERS)* vol. 1 417–420 (IEEE, 2005).
S42. Otieno, L. O. *et al.* A high bandwidth, high voltage amplifier for driving fast piezoelectric actuator-based nanopositioners used in atomic force microscopes. *J. Korean Phys. Soc.* **83**, 795–806 (2023).
